# OTULIN haploinsufficiency predisposes to environmentally directed inflammation

**DOI:** 10.3389/fimmu.2024.983686

**Published:** 2024-05-21

**Authors:** Frederik Staels, Leoni Bücken, Leana De Vuyst, Mathijs Willemsen, Erika Van Nieuwenhove, Margaux Gerbaux, Julika Neumann, Vanshika Malviya, Lize Van Meerbeeck, Jeason Haughton, Laura Seldeslachts, Mieke Gouwy, Kimberly Martinod, Greetje Vande Velde, Paul Proost, Lidia Yshii, Susan Schlenner, Rik Schrijvers, Adrian Liston, Stephanie Humblet-Baron

**Affiliations:** ^1^ Department of Microbiology, Immunology and Transplantation, Laboratory of Adaptive Immunology, KU Leuven, Leuven, Belgium; ^2^ Department of Microbiology, Immunology and Transplantation, Allergy and Clinical Immunology Research Group, KU Leuven, Leuven, Belgium; ^3^ VIB-KU Leuven Center for Brain and Disease Research, Leuven, Belgium; ^4^ Department of Imaging and Pathology, Biomedical MRI, KU Leuven, Leuven, Belgium; ^5^ Department of Microbiology, Immunology and Transplantation, Molecular Immunology, KU Leuven, Leuven, Belgium; ^6^ Department of Microbiology, Immunology and Transplantation, Molecular and Vascular Biology, KU Leuven, Leuven, Belgium; ^7^ Department of Neuroscience, KU Leuven, Leuven, Belgium; ^8^ Department of General Internal Medicine, University Hospitals Leuven, Leuven, Belgium; ^9^ Department of Pathology, University of Cambridge, Cambridge, United Kingdom

**Keywords:** OTULIN deficiency, inflammation, inborn errors in immunity, OTULIN-related autoinflammatory syndrome, OTULIN

## Abstract

Recently, OTULIN haploinsufficiency was linked to enhanced susceptibility to *Staphylococcus aureus* infections accompanied by local necrosis and systemic inflammation. The pathogenesis observed in haploinsufficient patients differs from the hyperinflammation seen in classical OTULIN-related autoinflammatory syndrome (ORAS) patients and is characterized by increased susceptibility of dermal fibroblasts to *S. aureus* alpha toxin-inflicted cytotoxic damage. Immunological abnormalities were not observed in OTULIN haploinsufficient patients, suggesting a non-hematopoietic basis. In this research report, we investigated an *Otulin^+/−^* mouse model after *in vivo* provocation with lipopolysaccharide (LPS) to explore the potential role of hematopoietic-driven inflammation in OTULIN haploinsufficiency. We observed a hyperinflammatory signature in LPS-provoked *Otulin^+/−^* mice, which was driven by CD64^+^ monocytes and macrophages. Bone marrow-derived macrophages (BMDMs) of *Otulin^+/−^* mice demonstrated higher proinflammatory cytokine secretion after *in vitro* stimulation with LPS or polyinosinic:polycytidylic acid (Poly(I:C)). Our experiments in full and mixed bone marrow chimeric mice suggest that, in contrast to humans, the observed inflammation was mainly driven by the hematopoietic compartment with cell-extrinsic effects likely contributing to inflammatory outcomes. Using an OTULIN haploinsufficient mouse model, we validated the role of OTULIN in the regulation of environmentally directed inflammation.

## Introduction

OTULIN has emerged as a key ubiquitin E3 ligase in the process of linear ubiquitination, a post-translational modification with essential regulatory roles in canonical NF-κB signaling and inflammatory responses (1–4). In 2016, the first description of otulipenic patients, caused by recessive loss-of-function (LOF) mutations in *OTULIN*, was published (5, 6), while more recently, a dominant negative mutation with a monoallelic heterozygous mutation has been reported (7). All patients presented with neonatal-onset life-threatening autoinflammation accompanied by fever, neutrophilic dermatitis, panniculitis, arthralgia/arthritis, lymphadenopathy, hepatosplenomegaly, gastrointestinal inflammation, and failure to thrive. This novel clinical entity was coined OTULIN-related autoinflammatory syndrome (ORAS). The precise molecular pathogenesis of how otulipenia results in a systemic inflammatory disorder is still under investigation, but current evidence suggests both hematopoietic and non-hematopoietic cell-specific effects of otulipenia (5, 6, 8–10). In the hematopoietic system, defective OTULIN function leads to an upregulation of linear ubiquitin-mediated signaling and spontaneous NF-κB activation in myeloid cells, but not in lymphocytes (5, 6). Otulipenic patients also displayed a remarkable activation of type I interferon signaling in whole blood because of proteasome dysregulation in OTULIN-deficient cells (11). Finally, the remission of all inflammatory symptoms after allogeneic stem cell transplantation in an ORAS patient is probably the most convincing evidence that hematopoietic cells are necessary for the clinical manifestation of ORAS (9). However, dermal fibroblasts of an ORAS patient were shown to be sensitized to TNF-induced cell death, and an increase in apoptotic cells was observed in skin lesions of an ORAS patient (9). In addition, selective knock-out of OTULIN in mouse keratinocytes, liver parenchymal cells, or intestinal epithelial cells caused an inflammatory skin disorder (10), severe hepatitis with increased risk of hepatocellular carcinoma (12), or susceptibility to colitis (13) respectively. Finally, knock-in mice expressing catalytically inactive OTULIN died during midgestation due to defective Wnt signaling and excessive endothelial cell death (14).

Recently, OTULIN haploinsufficiency has been associated with susceptibility to severe *Staphylococcus aureus* skin/pulmonary infections through a non-hematopoietic mechanism involving defective OTULIN-dependent caveolin 1 accumulation in dermal fibroblasts, which facilitated the cytotoxic damage mediated by the staphylococcal alpha toxin (1). Interestingly, no *S. aureus* infection was identified in the clinical course of disease in the probands of four kindreds, suggesting that other microbial triggers may trigger inflammatory disease (1, 15). A profound hematopoietic defect was not observed in three of these patients after *in vitro* characterization of peripheral blood mononuclear cells (PBMCs) through mass cytometry [cytometry by time-of-flight (CyTOF)] and RNA sequencing (1). However, *in vivo* stimulation by microbial stimuli could alter inflammatory responses in the presence of a genetic defect and reveal subtle changes that are not detected by *in vitro* assays. Therefore, we investigated the effect of microbial stimuli (lipopolysaccharide) on the immunological response in an *Otulin^+/−^* mouse model to explore the potential role of hematopoietic-driven inflammation in OTULIN haploinsufficiency.

## Methods

### Mice

All animal experiments were undertaken with the approval of the Ethics Committee on Animal Experiments (ECD number P008/2019). All mice have C57BL/6 background and were maintained under specific pathogen-free conditions in individually ventilated cages. *Otulin^+/−^* mice were a gift from Professor David Komander (MRC Laboratory of Molecular Biomedicine, Cambridge). Mice were allocated to experimental groups based on their genotype. Grouping of mice into cages was determined at weaning, but where possible, animals of equivalent age and gender were allocated to each experimental group. *Otulin^+/−^* or wild-type (*Wt*) littermate controls were injected with lipopolysaccharide (LPS; Sigma-Aldrich, Darmstadt, Germany) at a dose of 500 ng/kg or a sham [phosphate-buffered saline (PBS) 1× 100 μL] intraperitoneally 4 hours prior to sacrifice. In the bone marrow chimera (full and mixed) experiments, mice were only given LPS 500 ng/kg.

### Tissue and serum preparation

After 4 hours of *in vivo* stimulation, mice were euthanized. Blood was taken immediately after sacrifice by cardiac puncture. The blood was allowed to clot by leaving it at room temperature for 30 minutes. Thereafter, centrifugation at 2,000 × *g* for 10 minutes was performed, and serum was transferred to another Eppendorf tube for storage at −80°C. Spleen and bone marrow were harvested in complete Roswell Park Memorial Institute (RPMI) [RPMI 1640 (Life Technologies, Carlsbad, CA, USA) supplemented with 10% fetal bovine serum (FBS), penicillin/streptomycin, 5 μM β-mercaptoethanol, non-essential amino acids (GIBCO, Grand Island, NY, USA), and HEPES 10 mM (GIBCO)] and kept on ice until processing. Single-cell suspensions were prepared from the spleen and bone marrow by mechanical dissociation and red blood cell (RBC) lysis (155 mM NH_4_Cl, 12 mM NaHCO_3_, and 0.1 mM EDTA).

### Flow cytometry

For intracellular cytokine staining, cells were plated at 1–2 × 10^6^ cells/well in 96-well tissue-culture plates in complete RPMI containing either LPS (Sigma-Aldrich) 10 ng/mL or phorbol 12,13-dibutyrate (500 ng/mL, Bio-Techne, Minneapolis, MN, USA) and ionomycin (750 ng/mL, Bio-Techne), with brefeldin A (2 µg/mL, Bio-Techne) for 4 hours at 37°C.

Non-specific binding was blocked using 2.4G2 supernatant for mouse cells, and dead cells were labeled by fixable viability dye eFluor 780 (Thermo Fisher, Waltham, MA, USA). Cells were fixed and permeabilized using the eBioscience Foxp3 staining kit (eBioscience, San Diego, CA, USA). Spleen samples were stained overnight using the following antibodies: anti-IL-2 (JES6-5H4, rat IgG2b), anti-Ly6G (1A8, rat IgG2a), anti-IL-10 (JES5-16A3, rat IgG2b), anti-CD45 (30-F11, rat IgG2b), anti-CD45.1 (A20, mouse IgG2a), anti-CD45.2 (104, mouse IgG2a), anti-CD11b (M1/70, rat IgG2b), anti-Ly6C (HK1.4, rat IgG2c), anti-TNF-α (MP6-XT22, rat IgG1κ), anti-TCRγδ (GL3, Armenian hamster), anti-TCRβ (H57-597, Armenian hamster), anti-CD64 (X54-5/7.1, mouse IgG1), anti-pro-IL-1β (REA577, human IgG1), anti-CD11c (N418, Armenian hamster), anti-CD4 (GK1.5, rat IgG2b), anti-IL-6 (MP5-20F3, rat IgG1), anti-SiglecF (E50-2440, rat IgG2a), anti-IL-1β (B122, Armenian hamster), anti-IFN-γ (XMG1.2, rat IgG1), anti-CD8 (53-6.7, rat IgG2a), and anti-MHCII (M5/114.15.2, rat IgG2b). Bone marrow samples were stained for 1 hour at 4°C using following antibodies anti-CD45.1 (A20, mouse IgG2a), anti-CD45.2 (104, mouse IgG2a), anti-Sca1 (D7, rat IgG2a), anti-c-Kit (2B8, rat IgG2b), and anti-lineage (biotin anti-B220, anti-NK1.1, anti-CD3, anti-CD11c, anti-TER119, anti-CD11b, and anti-Gr1), followed by staining with secondary streptavidin (BV421, BioLegend, San Diego, CA, USA). Flow cytometry was performed on a BD FACSCanto II (bone marrow panel) or BD FACSymphony (spleen myeloid cytokine panel). Results were analyzed using FlowJo version 10.6.1 (Becton Dickinson, Ashland, OR, USA). Compensation for high-parameter flow panels was performed using AutoSpill (16).

### Mixed and full bone marrow chimeras

One day before transplantation, the drinking water of recipient mice was supplemented with 0.1 mg/mL enrofloxacin (Baytril^®^, Bayer, St. Louis, MO, USA). Bone marrow cells (1 × 10^6^) from *Wt* CD45.1 C57BL/6 mice and Otulin^+/−^ CD45.2 or *Wt* CD45.2 C57BL/6 littermate controls were resuspended in 100 μL PBS at a 1:1 ratio (only for mixed chimeras) and then injected retro-orbitally into 8–12-week-old sex- and age-matched γ-irradiated C57BL/6 CD45.1/CD45.2 recipients (given a total dose of 9.5 Gy). At 12 weeks after reconstitution, mice were used for experiments as described above.

### Bone marrow-derived macrophages and viability/stimulation assays

Bone marrow-derived macrophages (BMDMs) were generated from bone marrow cells harvested from tibias and femurs of *Otulin^+/−^* and *Wt* littermate C57BL/6 mice.

After collection, bones were flushed in complete RPMI (cRPMI). Cells were lysed in RBC lysis buffer and differentiated in cRPMI with 20 ng/mL recombinant mouse M-CSF (R&D Systems, Minneapolis, MN, USA) for 7 days. After differentiation, BMDMs were harvested and counted.

For cytokine analysis, BMDMs were seeded at a density of 1 × 10^5^ cells per well in a 96-well flat-bottom plate and stimulated with LPS (Sigma-Aldrich, 5 ng/mL) or Poly(I:C) (Sigma-Aldrich, 1.25 μg/mL) for 24 hours. Cells were centrifugated at 400 × *g* for 5 minutes, and the supernatant was transferred to another Eppendorf tube and stored at −80°C until analysis.

For viability assays, BMDMs were seeded at a density of 1 × 10^4^ cells per well in a 96-well flat-bottom plate. The next day, a stimulation medium containing TNF-α (PeproTech, Cranbury, NJ, USA, 300-01A) with or without cycloheximide (CHX; 50 μg/mL), and necrostatin-1 (Nec-1, 10 μM) was added for 6 or 24 hours. An MTS assay (ab197010) was immediately performed according to the manufacturer’s instructions. BMDMs denoted “unstimulated” in experiments indicate no exogenous stimulation of these cells after differentiation.

### Cytokine analysis

Cytokine/chemokine multiplex analysis was carried out using multiplex technology (MesoScale Discovery, Rockville, MD, USA). Serum and medium (from BMDM) samples were analyzed using the V-plex proinflammatory panel 1 (mouse) including the quantitative determination of IFN-γ, IL-1β, IL-2, IL-4, IL-5, IL-6, KC/GRO (CXCL-1), IL-10, IL-12p70, and TNF-α. The assay was performed according to the manufacturer’s instructions.

### Western blotting

BMDMs were plated at a density of 1 × 10^5^ cells per well in multiplicate in a 96-well U-bottom plate in cRPMI and rested overnight. The next day, a stimulation medium containing LPS 5 ng/mL was added during different time points (15 minutes, 30 minutes, and 60 minutes). Subsequently, cells were lysed in protein lysis buffer [50 mM Tris-HCl pH 7.5, 135 mM NaCl, 1.5 mM MgCl_2_, 1% Triton-X, 10% glycerol, 1× protease inhibitor (Pierce™ Protease Inhibitor, Thermo Fisher Scientific), and 1× phosphatase inhibitor (PhosSTOP, Roche, Basel, Switzerland)]. Protein concentrations were determined using a Bradford Protein Assay (Bio-Rad, Hercules, CA, USA). Protein lysate was denaturized in LDS (NuPAGE LDS sample buffer, Novex) and DTT (Bio-Rad) at 70°C for 10 minutes and was then loaded on a 4%–12% Bis-Tris polyacrylamide gel (Bolt™ Bis-Tris Plus, Thermo Fisher Scientific) in MOPS buffer (NuPAGE MOPS SDS running buffer, Novex). Separated proteins on the gel were transferred onto a methanol-activated polyvinylidene difluoride (PVDF) membrane (GE Healthcare, Little Chalfont, UK) in transfer buffer [10% methanol, 1× Tris/Glycine Buffer (Bio-Rad)] using the Tetra Blotting Module (Bio-Rad). The PVDF membrane was blocked in 5% milk Tris-buffered saline with Tween 0.1% (TBS-T) for 30 minutes at room temperature (RT) and then incubated overnight with primary antibody at 4°C followed by a wash with TBS-T and incubated with a secondary antibody for 1 hour at RT. The primary antibodies used for Western blotting were as follows: p-IκBα (14D4, #2859, CST, Danvers, MA, USA), IκBα (#9242, CST), p-P65 (93H1, #3033, CST), and actin (#4967, CST). Proteins were revealed using Amersham ECL prime Western Blotting Detection Reagent (GE Healthcare), and all blots were acquired using the G:box Chemi-XRQ.

### Statistics

All data reflect mean ± SEM, and all comparisons were statistically tested for experiments with ≥3 biological replicates within each group in GraphPad Prism 8.2.1, using unpaired two-tailed Student’s t-tests for comparing two experimental groups, one-way ANOVA for comparing three experimental groups, or two-way ANOVA to evaluate the mean of a quantitative variable according to the levels of two categorical variables (genotype and stimulation). With these statistical tests, significant differences are indicated and reflect the following p-values: *p < 0.05, **p < 0.01, and ***p < 0.001. Since mean fluorescence intensity (MFI) in absolute values was variable between independent experiments, all MFIs for a given cytokine were normalized to the MFI of the *Wt* sham-treated control. For the mixed bone marrow chimeras, the MFI was displayed as the ratio of CD45.2/CD45.1.

## Results

### LPS-provoked Otulin^+/−^ mice demonstrate an increased proinflammatory signature

**Figure 1:**
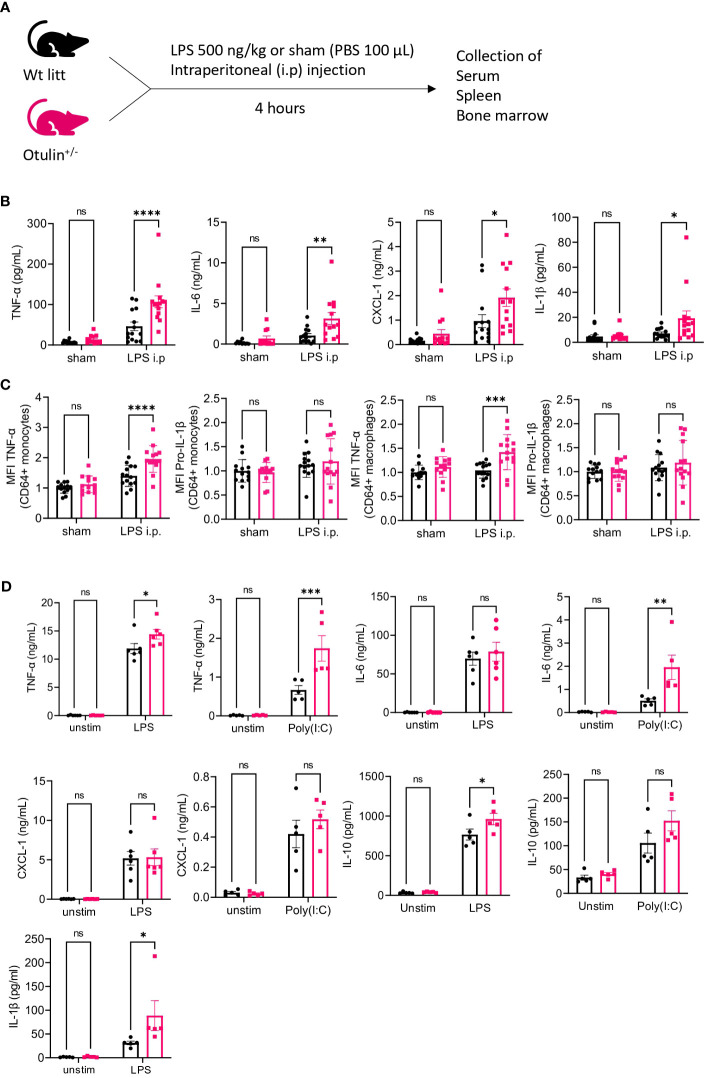
*Otulin^+/−^* mice have a myeloid-driven hyperinflammatory phenotype after *in vivo* LPS stimulation. **(A)** Experimental setup. *Wt*, wild type; litt, littermate. **(B)** Serum cytokine levels, n = 2 independent experiments, n = 13–14 mice in each group. **(C)** Normalized MFI of TNF-α and pro-IL-1β in TNF-α+ or pro-IL-1β+ CD64^+^ monocytes and macrophages to the mean of unstimulated sham-treated *Wt litt* control mice, n = 2 independent experiments, n = 13–14 mice in each group. **(D)** Cytokine analysis of supernatant of BMDMs stimulated with LPS 5 ng/mL or Poly(I:C) 1.25 μg/mL for 24 hours, n = 2 independent experiments; each dot represents the mean of a technical duplicate, n = 6 biological replicates. IL-1β was undetectable in the Poly(I:C) condition; hence, data are not shown. Statistics for all experiments were performed by two-way ANOVA and post-hoc Student’s t-tests. *p < 0.05, **p < 0.01, ***p < 0.001 and ****p < 0.001. Bars represent mean ± SEM. LPS, lipopolysaccharide; MFI, mean fluorescence intensity; BMDMs, bone marrow-derived macrophages. ns, not significant.

LPS was used as a microbial stimulus, as it is found in the outer membrane of gram-negative bacteria and stimulates innate immune cells by binding to toll-like receptor 4 (TLR4). Downstream receptor-associated proteins of TLR4 undergo linear ubiquitination by the linear ubiquitin assembly complex (LUBAC), which is counteracted by the deubiquitinase OTULIN (17). *Otulin^+/−^* and littermate control mice were challenged with an intraperitoneal injection of a sham (PBS 1×) or LPS for a duration of 4 hours to assess the production of inflammatory cytokines in the serum (**Figure 1A**). All proinflammatory cytokines (TNF-α, IL-6, CXCL-1, and IL-1β) were significantly increased in LPS-provoked *Otulin^+/−^* mice compared to littermate controls, whereas no significant differences were observed after the sham stimulus (**Figure 1B**).

### The hyperinflammatory signature is driven by myeloid cells

**Figure 2:**
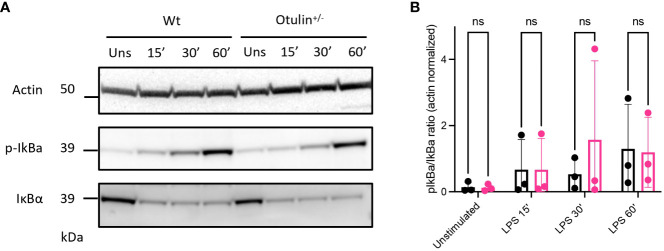
Canonical NF-κB signaling is unaffected by OTULIN haploinsufficiency. **(A)** Western blotting for p-IκBα and IκBα of LPS-stimulated (5 ng/mL) BMDMs, (representative data from three independent experiments). **(B)** Western blotting quantification of the three independent experiments. Unpaired Student’s t-test. LPS, lipopolysaccharide; BMDMs, bone marrow-derived macrophages. ns, not significant.

In a previous mouse model (5), a conditional knock-out of OTULIN in myeloid cells (*LysM-Cre*) resulted in autoinflammatory features resembling ORAS patients, whereas conditional knock-out in B (*Mb1-Cre*) or T cells (*CD4-Cre*) did not result in phenotypical or immunological abnormalities. To investigate whether the observed hyperinflammatory signature in LPS-provoked *Otulin^+/−^* mice was driven by the myeloid compartment, we assessed intracellular cytokine secretion of *in vitro* stimulated splenocytes in the different experimental groups (**Figures S1**, **S2**). Of note, the frequency of all immune subsets (myeloid and lymphoid) was identical between experimental groups (**Figure S3A**). We observed a significant increase of TNF-α and/or pro-IL-1β production (**Figure 1C**) with higher mean fluorescence intensity in the CD64^+^ monocytes and macrophages of LPS-provoked *Otulin^+/−^* mice compared to littermate controls, and we observed no significant differences after the sham stimulus. However, in all conditions, the percentages of TNF-α- and/or pro-IL-1β-producing CD64^+^ monocytes and macrophages remained identical in both *Otulin^+/−^* and *Wt* littermate controls (**Figure S3B**). In the lymphoid compartment (CD4^+^ and CD8^+^ cells), we found no differences within all groups in TNF-α, IL-2, or IFN-γ production (**Figure S3C**). Next, we differentiated BMDMs with recombinant mouse M-CSF for 7 days from *Otulin^+/−^* mice and littermate controls and challenged them *in vitro* with LPS or Poly I:C. BMDMs from *Otulin^+/−^* mice secreted more proinflammatory cytokines with significant differences in TNF-α, IL-6, IL-10, and IL-1β in one or both stimulation groups (**Figure 1D**). To further delineate the underlying molecular pathway driving this excessive inflammatory response, we investigated whether *Otulin^+/−^* BMDMs had increased canonical NF-κB signaling or were sensitized to TNF-induced cell death. However, we observed no differences in IκBα degradation (a surrogate marker for NF-κB activation) during LPS stimulation (**Figure 2** and **Figures S4A, B**) and cell viability after TNF-α stimulation with or without cycloheximide and inhibitors of necroptosis (necrostatin-1, RIPK1 inhibitor) (**Figures S4C, D**). Therefore, these experiments were not able to identify the precise mechanism contributing to the hyperinflammatory response in *Otulin^+/−^* myeloid cells.

### Hyperinflammation is driven by the hematopoietic cells with additional cell-extrinsic effects

**Figure 3:**
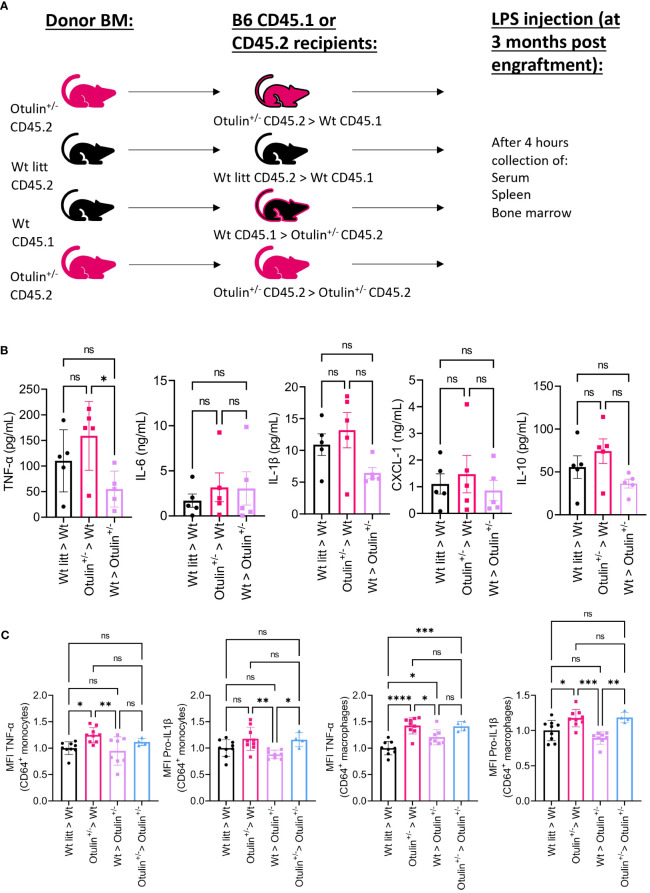
*Otulin* ^+/−^ > *Wt* chimeras demonstrate a hematopoietic-driven inflammation. **(A)** Experimental setup. BM, bone marrow; *Wt*, wild type; litt, littermate. **(B)** Serum cytokine levels, n = 1 independent experiment, n = 5 mice in each group. **(C)** Normalized MFI of TNF-α and pro-IL-1β in TNF-α+ or pro-IL-1β+ CD64^+^ monocytes and macrophages to the mean MFI in *Wt > Wt litt* control mice; pooled data from two independent experiments, n = 4–9 mice in each group. Statistics for all experiments were performed by one-way ANOVA. *p < 0.05, **p < 0.01, ***p < 0.001 and ****p < 0.001. Bars represent mean ± SEM. MFI, mean fluorescence intensity. ns, not significant.

**Figure 4:**
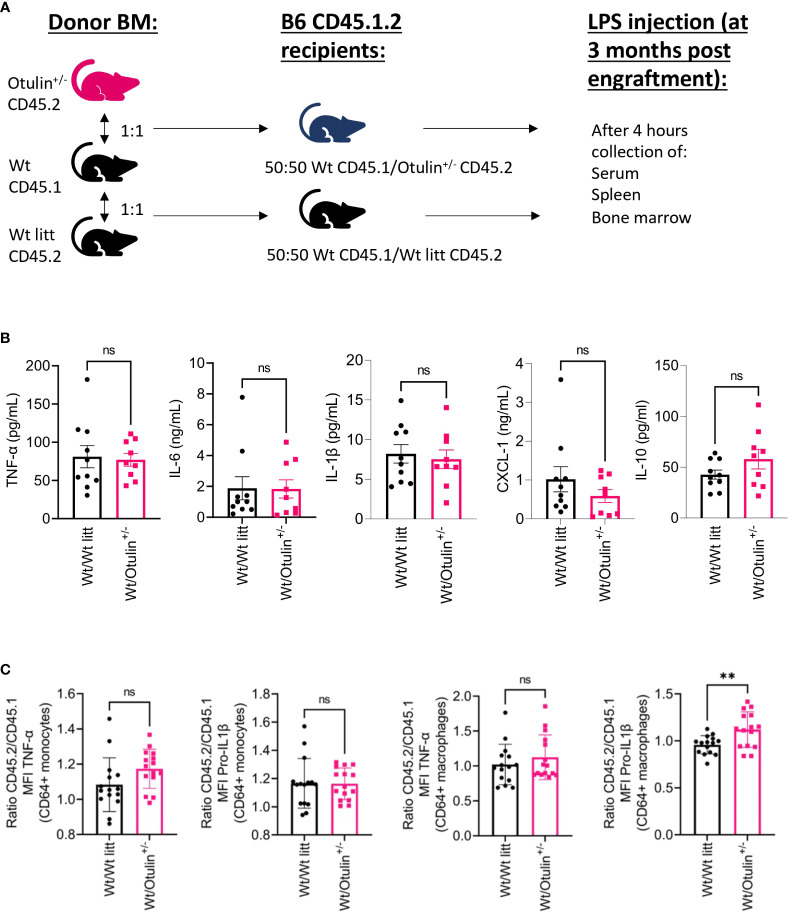
Cell-intrinsic effects contribute to the inflammatory outcome in mixed BM chimera. **(A)** Experimental setup. BM, bone marrow; *Wt*, wild type; litt, littermate. **(B)** Serum cytokine levels, n = 2 independent experiments, n = 9–10 mice in each group. **(C)** Normalized ratio (fold change) of MFI of TNF-α and pro-IL-1β in TNF-α+ or pro-IL-1β+ CD64^+^ monocytes and macrophages from *Otulin^+/−^* or *Wt litt* cells to Wt cells; pooled data from three independent experiments, n = 15 mice in each group. Statistics for all experiments were performed by unpaired Student’s t-test. **p < 0.01. Bars represent mean ± SEM. MFI, mean fluorescence intensity. ns, not significant.

To determine the contribution of the hematopoietic and non-hematopoietic compartments, a bone marrow chimera model was set up and provoked *in vivo* with LPS (**Figure 3A**). Engraftment was seen after 3 months in all mice (data not shown). Serum TNF-α was significantly increased in *Otulin^+/−^ > Wt* compared to *Wt > Otulin^+/−^* mice, and for the other serum proinflammatory cytokines, mean values were the highest in the *Otulin^+/−^ > Wt* group (**Figure 3B**). Correspondingly, similar and significant findings were found in the levels of TNF-α produced by CD64^+^ monocytes and macrophages in the *Otulin^+/−^ > Wt* group compared to *Wt > Otulin^+/−^* and *Wt littermate > Wt*. Interestingly, a slight contribution from the radiation-resistant cells cannot be excluded, as macrophages from *Otulin^+/−^* recipients receiving *Wt* BM expressed higher TNF-α than the *Wt littermate > Wt* control group. For IL-1β, while the serum did not show significant differences between the different groups, myeloid cells from *Otulin^+/−^* donor origin displayed an increased IL-1β secretion (**Figure 3C**). To evaluate if there was a cell-intrinsic effect, a mixed bone marrow chimera model (50:50) was evaluated (**Figure 4A**, **Figures S6A, B**). In the group of *Wt/Otulin* ^+/−^ (50:50), 50% of total cells were OTULIN haploinsufficient, as OTULIN haploinsufficient cells did not carry a proliferative (dis)advantage to *Wt* cells after engraftment (**Figure S6B**). Comparing *Wt/Otulin* ^+/−^ (50:50) and *Wt/Wt* littermate (50:50), no difference in serum cytokine levels was observed after the LPS challenge, illustrating an absence of systemic hyperinflammation (**Figure 4B**). To evaluate cell-intrinsic effects, intracellular cytokine production was compared in Wt and *Otulin^+/−^* CD64^+^ monocytes and macrophages. There, increased cytokine secretion in *Otulin^+/−^* cells was present compared to the *Wt* cells, although not to a sufficient level to be detected in the serum (**Figure 4C** and **Figure S5B**). Hence, *Otulin^+/−^* mice display a hyperinflammatory signature, which is largely driven by hematopoietic cells, while contributing cell-extrinsic effects cannot be excluded.

## Discussion

OTULIN haploinsufficiency has recently been linked to susceptibility to severe *S. aureus* infections (1), phenotypically differing from otulipenic patients who present with life-threatening ORAS (5, 9). These phenotypical differences can be explained by a distinct pathogenesis (1, 5). Whereas ORAS patients display enhanced myeloid-driven inflammation, type I IFN responses, and increased susceptibility to TNF-driven cell death (5, 6, 9, 11), OTULIN haploinsufficient patients [including 5p− syndrome (Cri Du Chat) patients representing a phenocopy of OTULIN haploinsufficiency] have no overt immunological abnormalities but do have an increased susceptibility to cytotoxic effects of the *S. aureus* alpha toxin based on increased OTULIN-dependent caveolin 1 accumulation in dermal fibroblasts (1). In four kindreds, systemic inflammation and local necrosis occurred without a documented *S. aureus* infection (1, 15). Furthermore, in none of the otulipenic patients, severe *S. aureus* infections were explicitly reported (5, 9, 11). This questions whether stimuli other than *S. aureus* could be provocative of excessive systemic inflammation.

To determine whether OTULIN haploinsufficient individuals may be at enhanced risk of alternative inflammatory triggers, we studied *Otulin^+/−^* mice subjected to *in vivo* stimulation with LPS, a well-defined TLR4 stimulus. OTULIN plays important regulatory roles downstream of TLR4 by deubiquitination of receptor-associated proteins (17). Previous reports postulated that *Otulin^+/−^* mice were healthy (5) but that no immunological data were available and they were not subjected to environmental triggers. Indeed, we observed no immunophenotypical differences between *Otulin^+/−^* and littermate controls at baseline in terms of leukocyte subset frequencies or cytokine production after *in vitro* stimulation. However, upon *in vivo* stimulation with LPS, the myeloid cells of *Otulin^+/−^* mice (CD64^+^ monocytes and macrophages) displayed higher production of TNF-α and/or pro-IL-1β. Correspondingly, these proinflammatory cytokines (together with IL-6 and CXCL-1) were also elevated in the serum. The most striking difference was found for TNF-α, which is the main driver of inflammation in ORAS patients, as previous reports demonstrated remission of inflammatory symptoms using anti-TNF treatment in human subjects and mixed bone marrow chimera mice (50:50 *Wt*/*CreERT-Otulin^LZ/FL^*) (5, 9). In addition, a recent case report described a successful response after anti-TNF treatment in an OTULIN haploinsufficient patient suffering from severe fasciitis and skin necrosis (15). Further, our observation of myeloid-driven inflammation in OTULIN haploinsufficiency was supported by the characterization of BMDMs from *Otulin^+/−^* and littermate control mice. *Otulin^+/−^* BMDMs produced significantly more proinflammatory cytokines in response to LPS and/or Poly(I:C) *in vitro* stimulation.

The underlying molecular mechanism of hyper-responsivity in *Otulin* heterozygous mice was not resolved in our study, as NF-κB activation and sensitization to TNF-induced cell death in BMDMs were comparable between *Otulin^+/−^* and *Wt* littermate controls. Recently, enhanced type I IFN responses have been demonstrated in ORAS patients (11) and mice expressing catalytically inactive OTULIN with co-deletion of caspase 8 and RIPK3 (*Otulin^C129A/C129A^ Ripk3^−/−^ Casp8^−/−^*) (14). In addition, specific OTULIN deficiency in myeloid cells identified a RIPK3-dependent pathway that was acting as an NLRP3 inflammasome activator promoting IL-1β secretion in macrophages (18). Hence, this could be a potential mechanism in OTULIN haploinsufficiency requiring validation. The observation of a myeloid-driven phenotype is consistent with the known roles of OTULIN in specific immune cells (5). Studies in mice lacking OTULIN in myeloid cells (*LysM-Cre*) showed an ORAS phenotype, while mice lacking OTULIN in B (*Mb1-Cre*) and T cells (*CD4-Cre*) were healthy without signs of inflammation (5). Current evidence strongly points toward a hematopoietic-driven inflammation in ORAS patients, as allogeneic stem cell transplantation in one patient resulted in the complete resolution of all inflammatory symptoms (9). However, a contribution of non-hematopoietic effects involving sensitization to TNF-dependent or independent cell death to the phenotype is supported by numerous studies in OTULIN transgenic mice and dermal fibroblasts of ORAS patients (8–10). In our mouse model, the role of the hematopoietic compartment was supported by the observations in full bone marrow chimera mice, where *Otulin^+/−^* > *Wt* mice had higher (non-significant) serum cytokine levels compared to *Wt* > *Otulin^+/−^* and *Wt* littermate > *Wt* mice. Further, on a cellular basis, mice receiving *Otulin^+/−^* bone marrow displayed higher secretion of both TNF-α and pro-IL-1β by their CD64+ myeloid cells. However, we could not rule out the role of additional cell-extrinsic mechanisms to further contribute to the inflammatory outcome given the absence of systemic hyperinflammation in the serum of the mixed chimera (*Wt/Otulin^+/−^* 50:50) mice, while on a single cell level, *Otulin^+/−^* myeloid cells displayed higher inflammatory cytokine secretion. On the contrary, the overall systemic decrease of inflammation in these mixed chimera mice (*Wt/Otulin^+/−^* 50:50) could be still attributed to suppressive signals coming from extrinsic *Wt* cells or could be the result of insufficient *Otulin^+/−^* cell number to contribute to a systemic inflammatory effect. Further experiments are required to test these different hypotheses.

While OTULIN haploinsufficiency in humans is linked to susceptibility to *S. aureus* alpha toxin-inflicted cytotoxic damage in non-hematopoietic cells, our research in an *Otulin^+/−^* mouse model highlights the role of OTULIN in the control of environmentally directed inflammatory responses driven by myeloid cells and thereby contributes to the knowledge of OTULIN function. Interestingly, these two observations are not incompatible, as in both cases, the baseline study of myeloid cells did not show any difference from the healthy myeloid compartment. In addition, controlled triggered external challenges cannot obviously be tested in patients (an overview of human and transgenic mouse genotype and phenotype is shown in **Table 1**). The Otulin–LUBAC complex has been involved in anti-microbial response (22). While ORAS patients have not been documented with susceptibility to infection, OTULIN haploinsufficiency is dominated by a high inflammatory response after infectious external triggers (*S. aureus* in humans and LPS in mice). Interestingly, predisposition to additional infectious diseases with an increased susceptibility to *Salmonella* infection in mice harboring a specific *Otulin* defect in intestinal epithelial cells has also been recently reported (13). The contribution of environmental triggers to disease manifestation in the context of a given genetic defect was demonstrated in other mouse models (23, 24). *Rela^+/−^* mice, used as a model for RELA haploinsufficient patients, developed normally and only demonstrated cutaneous ulceration when provoked by intradermally injected TNF (23). Recently, iRHOM2 deficiency (encoded by *Rhbdf2*) was described in human subjects presenting with recurrent pulmonary and intestinal infections (24). *Rhbdf2^−/−^* mice were healthy without pathological abnormalities but showed increased susceptibility to *Pseudomonas aeruginosa* and *Citrobacter rodentium* infections (24). Hence, our model fits in this category of environmentally triggered disease. Several limitations to our research need to be addressed. First, a molecular mechanism driving the inflammatory response was not identified. We assessed canonical NF-κB activation and sensitization to TNF-induced cell death in BMDMs, but we found no differences between *Otulin^+/−^* and littermate control BMDMs. A more in-depth investigation using RNA sequencing of (un)stimulated BMDMs can provide more details on activated pathways and reveal subtle changes in NF-κB activation, cell death pathways, or type I IFN responses. Also, the presence/abundance of linear ubiquitin chains and expression of LUBAC was not evaluated in splenocytes or non-hematopoietic cells; hence, no conclusions of the effect of OTULIN haploinsufficiency on linear chain formation and LUBAC expression could be made. However, as OTULIN haploinsufficient patients were shown to have increased levels of linear ubiquitin in fibroblasts, a similar effect would be expected in mice (1). Second, our findings in LPS-provoked *Otulin^+/−^* compared with littermate controls were not fully reproduced in the full bone marrow chimera model on serum cytokine level where *Otulin^+/−^* > *Wt* transplants only had a significant increase in TNF-α production by myeloid cells compared to *Wt* > *Otulin^+/−^* and *Wt* littermate > *Wt* mice. These findings suggest an additional contribution of—yet undefined—cell-extrinsic effects to the inflammatory outcome. Finally, no overt phenotype was observed in *Otulin^+/−^* mice, possibly because of the short exposure (4 hours) to LPS. A longer duration of administration of multiple LPS pulses would be an option to explore phenotypical differences between *Otulin^+/−^* and littermate controls. However, the primary objective of this research was to assess inflammatory responses based on an immunological evaluation irrespective of the presence or absence of an overt clinical phenotype.

**Table 1:** Genotype–phenotype relation of *OTULIN* in human and mouse transgenic models (models with co-deletions not shown).

Species	Genotype	Phenotype	Reference
Human			
Otulipenia	AR or CH: L272P, Y244C, G174DfsX2, G281R, c.864 + 2T > C, M86I;W167S	OTULIN-related autoinflammatory syndrome	(5, 6, 9, 19, 20)
OTULIN autosomal dominant mutation	AD: C129S	OTULIN-related autoinflammatory syndrome	(7)
OTULIN haploinsufficiency	AD: N341D, P254S, D246V R263Q, E95X, D268TfsX5, 5p− syndrome	Susceptibility to *Staphylococcus aureus* alpha toxin-inflicted cytotoxic damage	(1, 15)
Mouse			
Full deletion or knock-in of catalytically inactive OTULIN	*Otulin^C129A/C129A^*, *Otulin^W96A/W96A^*, *Otulin^D336E/D336E^*	Lethal (E10.5-14); impaired Wnt signaling, abnormal yolk sac vasculature; excessive endothelial cell death in yolk sac and placenta (C129A)	(14, 21)
Inducible full deletion	*Otulin^LacZ/FL^ERT2-Cre*	Die within a day after tamoxifen treatment	(5)
Inducible catalytic inactive	*Otulin^iC129A/C129A^ERT2-Cre*	Systemic inflammation; tissue degeneration in bone marrow, thymus, liver, small intestine, and heart	(14)
Endothelial-specific deletion	*Otulin^iC129A/C129A^Cdh5-Cre*	Disruption of the yolk sac vasculature at E11.5, preceded by extensive apoptosis at E10.5	(14)
Keratinocyte-specific deletion	*Otulin^FL/FL^Keratin14-Cre*	Inflamed skin lesions, verrucous carcinoma	(10)
Intestinal epithelial cell-specific deletion	*Otulin^FL/FL^Villin-Cre*	Susceptibility to induced colitis (after DSS, TNF-α, and *Salmonella*)	(13)
Hepatocyte-specific deletion	*Otulin^FL/Fl^Alfp-Cre*	Hepatitis, liver fibrosis, hepatocellular carcinoma	(12)
B cell-specific deletion	*Otulin^LacZ/FL^Mb1-Cre*	No phenotype	(5)
T cell-specific deletion	*Otulin^LacZ/FL^CD4-Cre*	No phenotype	(5)
Myeloid-specific deletion	*Otulin^LacZ/FL^LysM-Cre*	Systemic inflammation and autoimmunity	(5)
OTULIN haploinsufficiency	*Otulin^+/−^*	Microbial (LPS)-provoked hyperinflammation driven by myeloid cells	

AR, autosomal recessive; CH, compound heterozygous; AD, autosomal dominant; LPS, lipopolysaccharide.

## Data availability statement

The original contributions presented in the study are included in the article/**Supplementary Material**. Further inquiries can be directed to the corresponding author.

## Ethics statement

All animal experiments were undertaken with the approval of the Ethics Committee on Animal 105 Experiments (ECD number P008/2019).

## Author contributions

Conceptualization: FS, AL, and SH-B. Formal analysis: FS. Investigation: FS, MW, EN, MGe, JN, LS, MGo, KM, GV, PP, SS, and RS. Writing – original draft: FS, RS, SH-B, and AL. Writing- review and editing: all. Visualization: FS. Supervision: RS, AL, and SH-B. All authors contributed to the article and approved the submitted version.

## Supplementary material

Supplementary Figure 1 Gating strategy used for identifying lymphoid (T cells) and myeloid subsets (CD64^+^ monocytes and macrophages) in spleen.

Supplementary Figure 2 Gating intracellular cytokine secretion after in vitro stimulation of lymphoid (T cells) and myeloid subsets (CD64^+^ monocytes and macrophages). **(A)** Representative flow plots and histogram of TNF-α and Pro-IL1β expression in myeloid cells from Otulin ^+/-^ mice and Wt litt control mice. For myeloid activation LPS 10 ng/mL with Brefeldin A 2 µg/ml were used for 4 hours. **(B)** Representative flow plots of TNF-α and IFN-γ and IL-2 expression in CD4^+^ and CD8^+^ T cells from Otulin ^+/-^ mice and Wt litt control mice. For T cell activation Phorbol 12,13-dibutyrate (500 ng/mL) with Brefeldin A 2 µg/mL was used for 4 hours.

Supplementary Figure 3
*Leukocyte frequencies and percentage of cytokine secreting cells* **(A)** Leukocyte frequencies for all subsets as percentage from live CD45^+^ cells: CD4^+^ T cells (live CD45^+^TCRαβ^+^CD4^+^), CD8^+^ T cells (live CD45^+^TCRαβ^+^CD4^+^), CD64^+^ monocytes (live CD45^+^CD11b^+^Ly6G^-^MHCII^high-^Ly6C^+^CD64^+^), CD64^+^macrophage (live CD45^+^CD11b^+^Ly6G^-^MHCII^high-^Ly6C^-^CD64^+^), neutrophils (live CD45^+^CD11b^+^Ly6G^+^) and dendritic cells (live CD45^+^CD11b^+^Ly6G^-^MHCII^high-^CD11c^+^). **(B)** Percentages of TNF-α and pro-IL1β producing cells from CD64^+^ monocytes and macrophages. **(C)**. MFI of IL-2, TNF-α and IFNγ of CD4^+^ and CD8^+^ cells. n=2 independent experiments, n=13-14 mice in each group. Statistics for all experiments were done by two-way ANOVA and *post hoc* Student t-tests. *p < 0.05, **p < 0.01, and ***p < 0.001. Bars represent mean ± SEM.

Supplementary Figure 4
*Canonical NF-κB signaling and TNF-dependent cell death is unaffected by OTULIN haploinsufficiency* **(A)** Replicate of the Western blot for p-IκBα, IκBα of LPS stimulated (5 ng/mL) BMDMs, **(B, C)** Relative cell viability after 6 hours **(B)** and 24 hours **(C)** of stimulation of BMDMs by MTS test, normalized to untreated conditions. TNF-α 100 ng/ml; CHX 50μg/mL; Nec-1, 10μM. Each dot represents an individual experiment, as biological repeat (n=3) each experiment was performed with up to 6 technical replicates. Bars represent mean ± SEM.

Supplementary Figure 5 Gating strategy in BM chimera and cytokine expression in mixed BM mice. **(A)** Gating strategy in BM chimera used for identifying lymphoid (T cells) and myeloid subsets (CD64^+^ monocytes and macrophages) in spleens according to CD45.1 or CD45.2 genotype in BM chimeras. **(B)**. Comparison within the same mouse of the MFI of TNF-α and pro-IL1β in TNF-α+ or pro-IL1β+ CD64^+^ monocytes and macrophages, pooled data from 3 independent experiments, n=15 mice in each group. Statistics for all experiments were done by paired Student t-test. *p < 0.05, **p < 0.01, and ***p < 0.001. Bars represent mean ± SEM.

Supplementary Figure 6
*Assessment of mouse chimerism after mixed bone marrow transplantation*. **(A)**. Gating strategy for bone marrow samples **(A)** and input ratio determination based on relative CD45.2 and CD45.1 counts from live CD45^+^ and progenitor cells (live CD45^+^ Lineage ^–^ cKit^+^ Sca-1^+^) cells. **(B)**. Chimerism assessment 3 months after transplantation in lymphoid (T cells) and myeloid subsets (CD64^+^ monocytes and macrophages) in spleen, n=2 independent experiments, n=9-10 mice in each group. Bars represent mean ± SEM.
