# Supplementary figures and images for: OTULIN haploinsufficiency predisposes to environmentally directed inflammation
Source: Front Immunol. 2024 May 21;15:983686. doi: 10.3389/fimmu.2024.983686 (PMC11140568; doi:10.3389/fimmu.2024.983686)

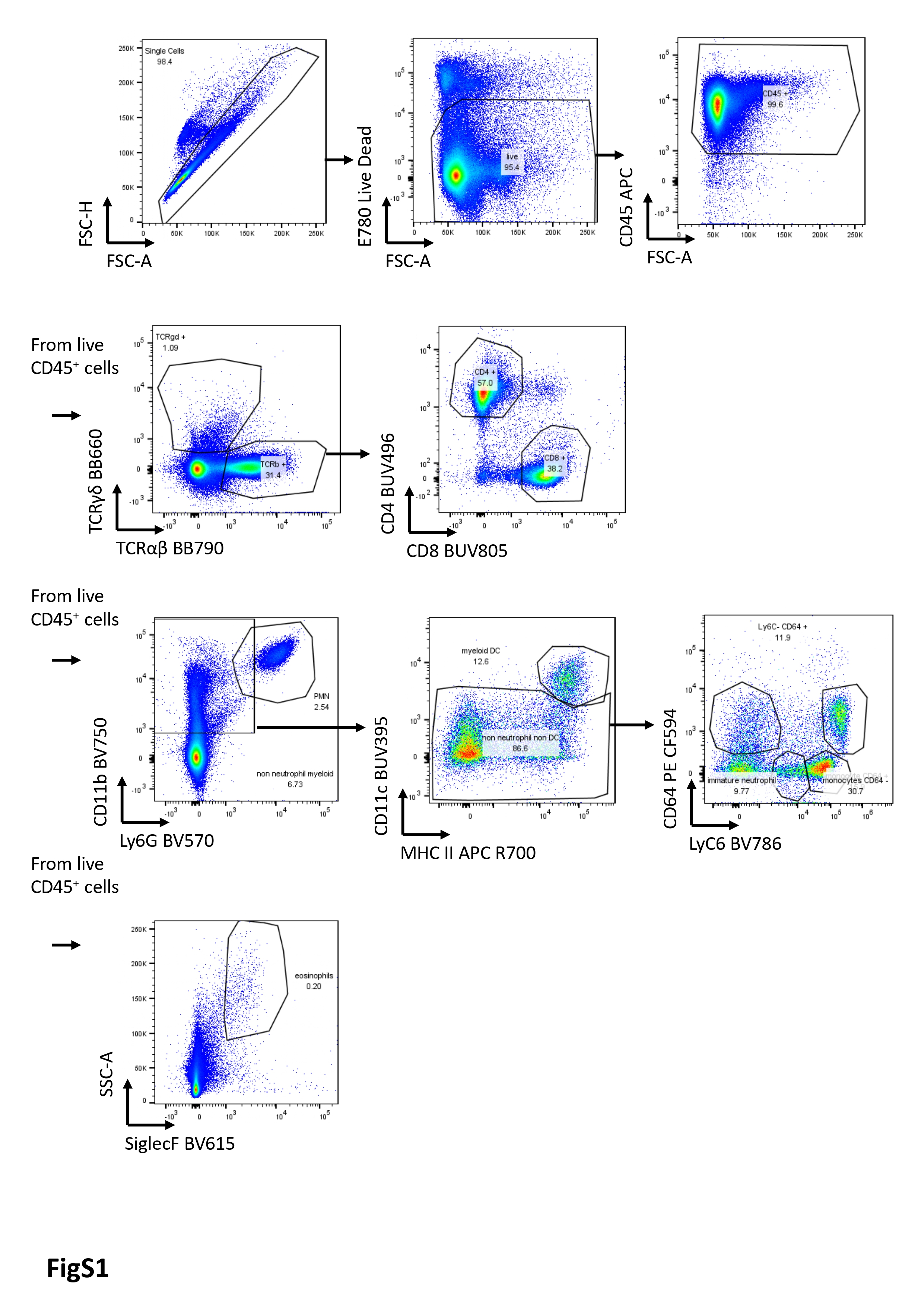

Supplement: Supplementary Figure 1 — Gating strategy used for identifying lymphoid (T cells) and myeloid subsets (CD64+ monocytes and macrophages) in spleen. [file Image_1.jpg]

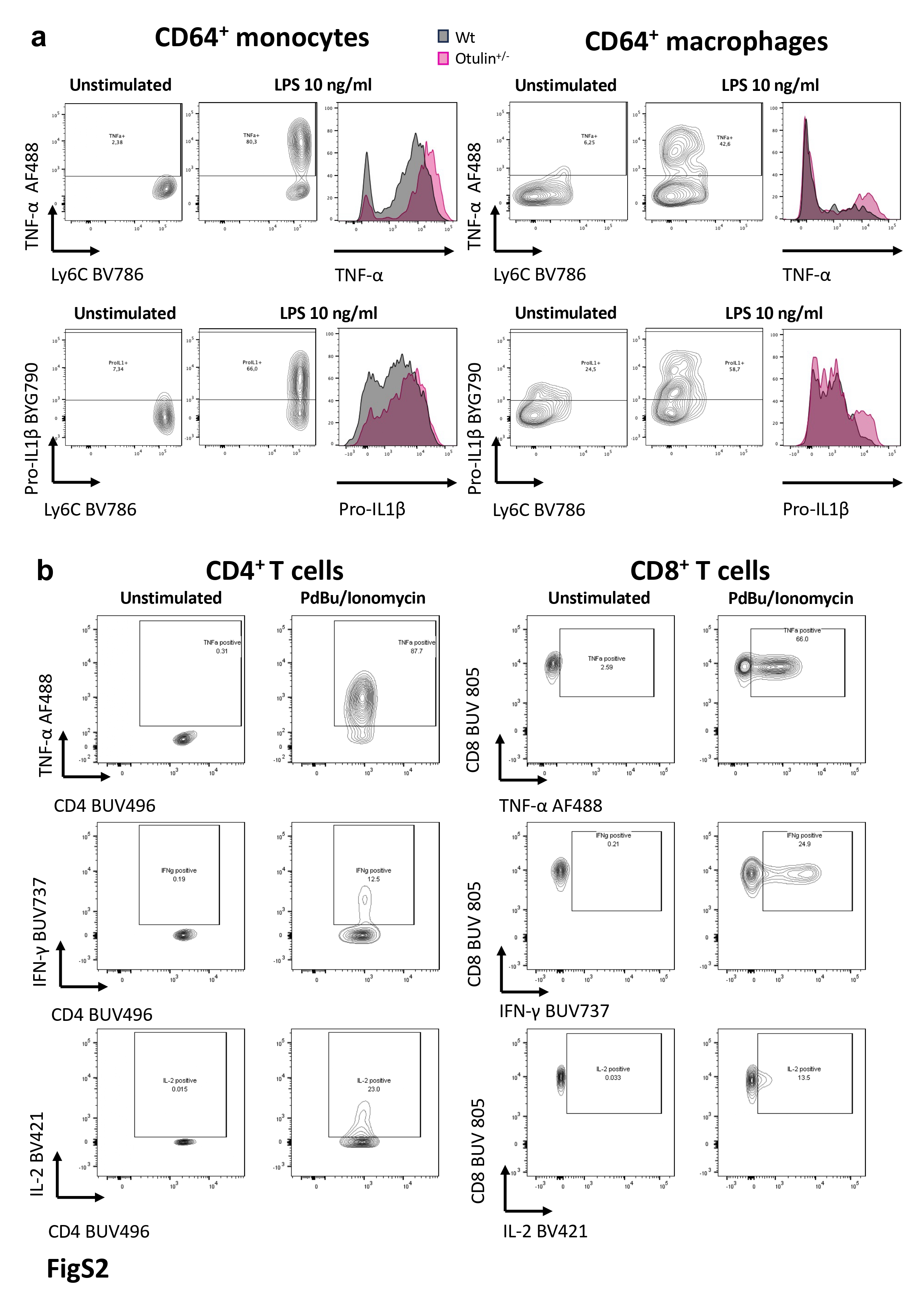

Supplement: Supplementary Figure 2 — Gating intracellular cytokine secretion after in vitro stimulation of lymphoid (T cells) and myeloid subsets (CD64+ monocytes and macrophages). (A) Representative flow plots and histogram of TNF-α and Pro-IL1β expression in myeloid cells from Otulin +/- mice and Wt litt control mice. For myeloid activation LPS 10 ng/mL with Brefeldin A 2 µg/ml were used for 4 hours. (B) Representative flow plots of TNF-α and IFN-γ and IL-2 expression in CD4+ and CD8+ T cells from Otulin +/- mice and Wt litt control mice. For T cell activation Phorbol 12,13-dibutyrate (500 ng/mL) with Brefeldin A 2 µg/mL was used for 4 hours. [file Image_2.jpg]

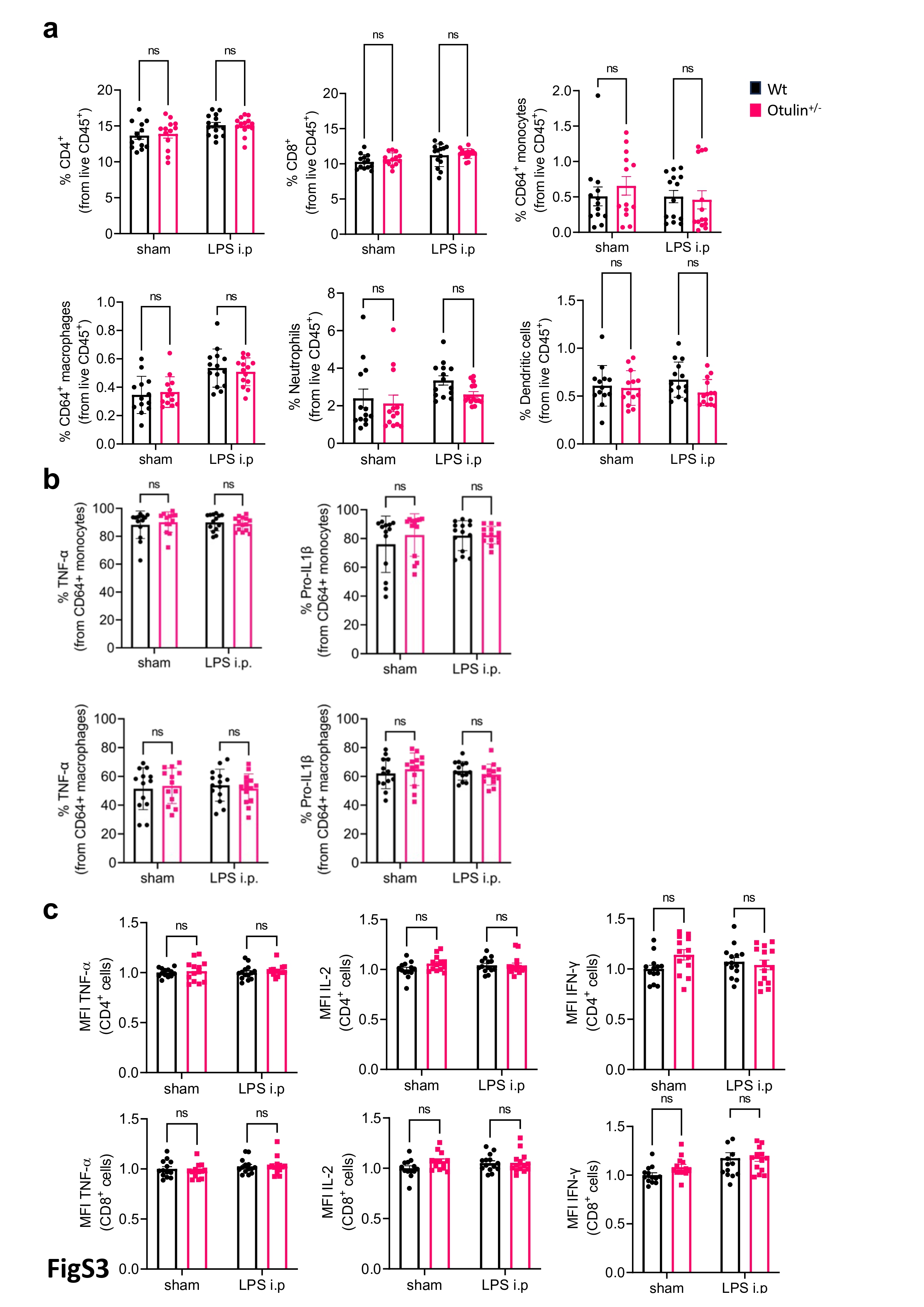

Supplement: Supplementary Figure 3 — Leukocyte frequencies and percentage of cytokine secreting cells (A) Leukocyte frequencies for all subsets as percentage from live CD45+ cells: CD4+ T cells (live CD45+TCRαβ+CD4+), CD8+ T cells (live CD45+TCRαβ+CD4+), CD64+ monocytes (live CD45+CD11b+Ly6G-MHCIIhigh-Ly6C+CD64+), CD64+macrophage (live CD45+CD11b+Ly6G-MHCIIhigh-Ly6C-CD64+), neutrophils (live CD45+CD11b+Ly6G+) and dendritic cells (live CD45+CD11b+Ly6G-MHCIIhigh-CD11c+). (B) Percentages of TNF-α and pro-IL1β producing cells from CD64+ monocytes and macrophages. (C). MFI of IL-2, TNF-α and IFNγ of CD4+ and CD8+ cells. n=2 independent experiments, n=13-14 mice in each group. Statistics for all experiments were done by two-way ANOVA and post hoc Student t-tests. *p < 0.05, **p < 0.01, and ***p < 0.001. Bars represent mean ± SEM. [file Image_3.jpg]

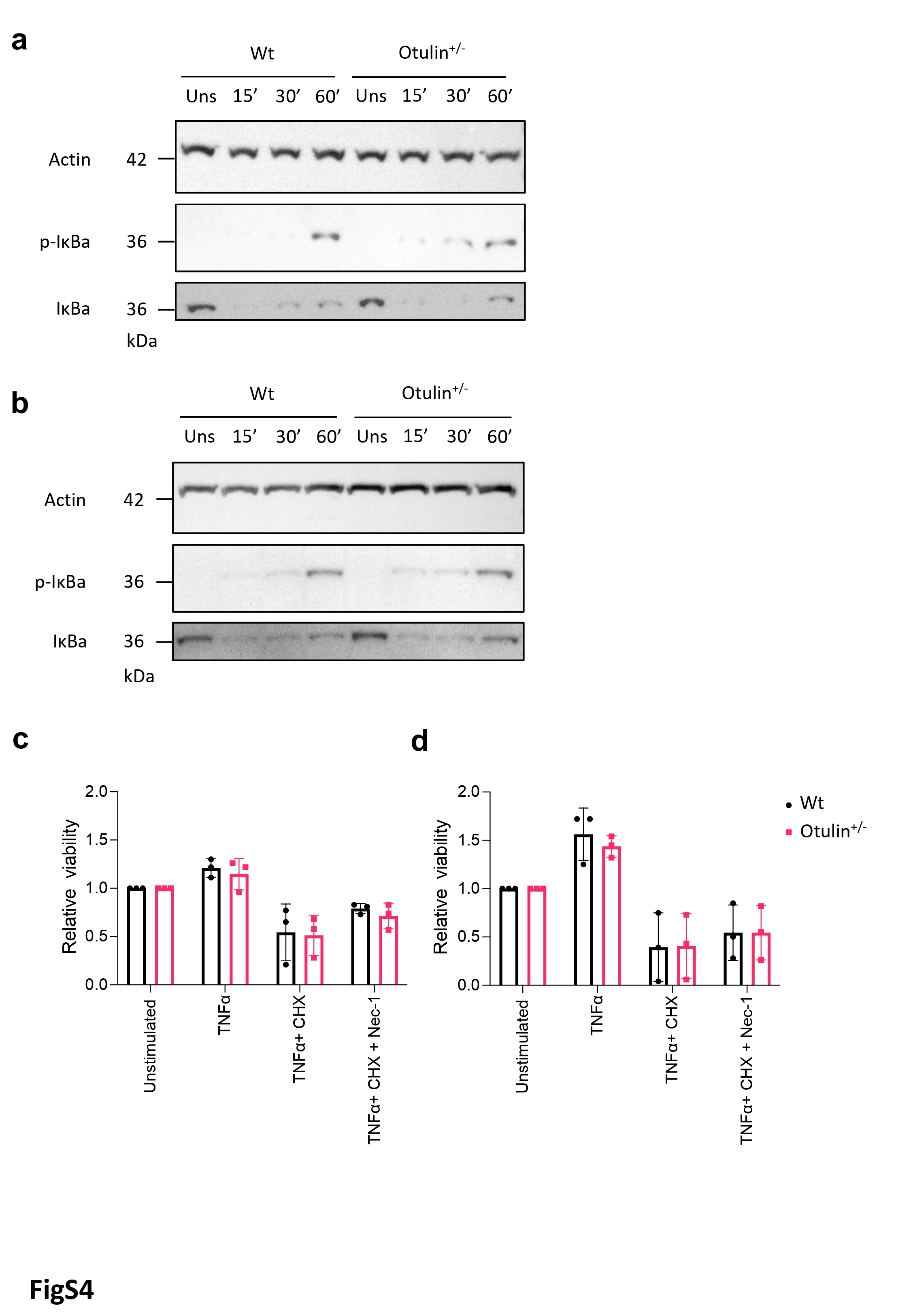

Supplement: Supplementary Figure 4 — Canonical NF-κB signaling and TNF-dependent cell death is unaffected by OTULIN haploinsufficiency (A) Replicate of the Western blot for p-IκBα, IκBα of LPS stimulated (5 ng/mL) BMDMs, (B, C) Relative cell viability after 6 hours (B) and 24 hours (C) of stimulation of BMDMs by MTS test, normalized to untreated conditions. TNF-α 100 ng/ml; CHX 50μg/mL; Nec-1, 10μM. Each dot represents an individual experiment, as biological repeat (n=3) each experiment was performed with up to 6 technical replicates. Bars represent mean ± SEM. [file Image_4.jpg]

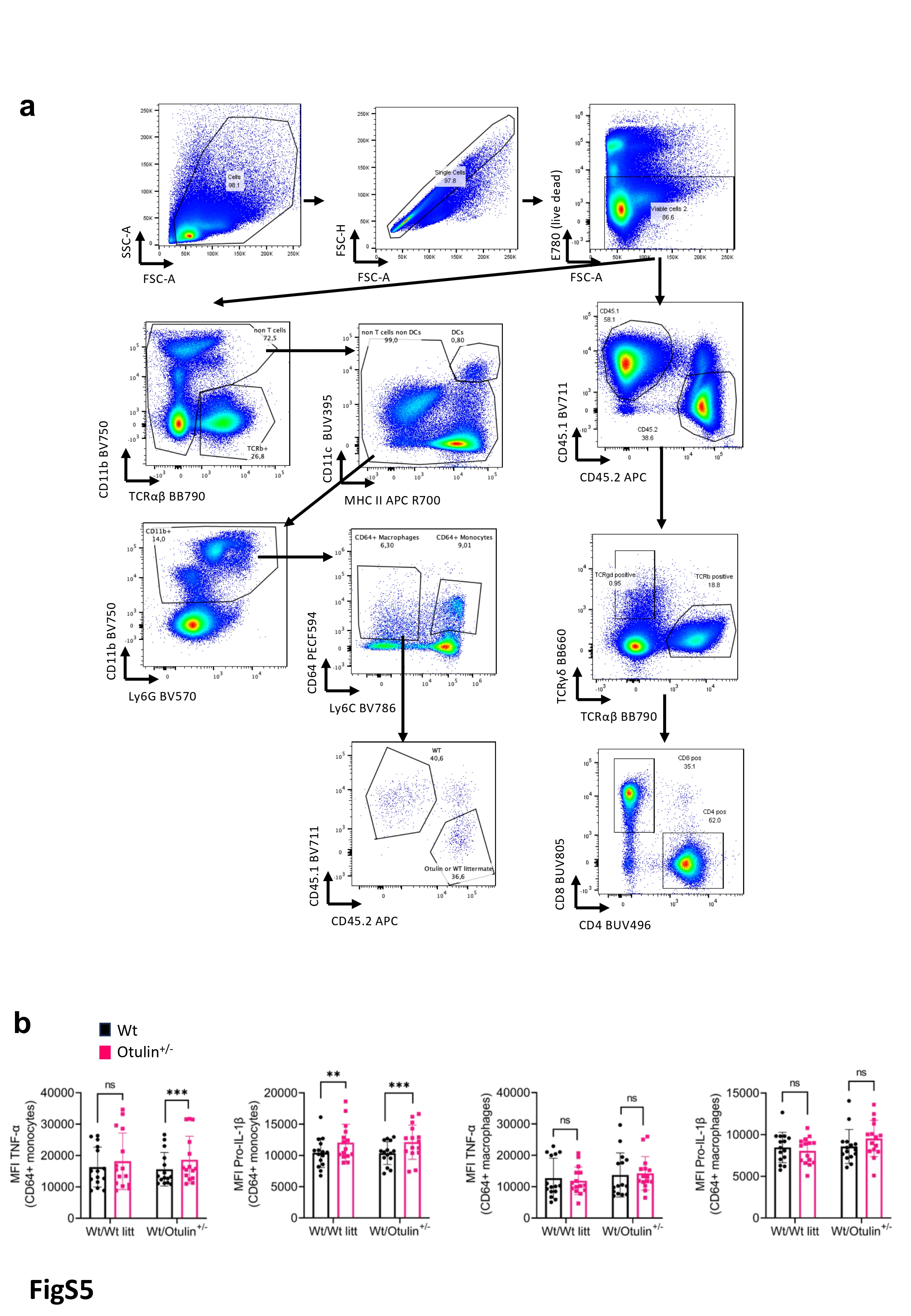

Supplement: Supplementary Figure 5 — Gating strategy in BM chimera and cytokine expression in mixed BM mice. (A) Gating strategy in BM chimera used for identifying lymphoid (T cells) and myeloid subsets (CD64+ monocytes and macrophages) in spleens according to CD45.1 or CD45.2 genotype in BM chimeras. (B). Comparison within the same mouse of the MFI of TNF-α and pro-IL1β in TNF-α+ or pro-IL1β+ CD64+ monocytes and macrophages, pooled data from 3 independent experiments, n=15 mice in each group. Statistics for all experiments were done by paired Student t-test. *p < 0.05, **p < 0.01, and ***p < 0.001. Bars represent mean ± SEM. [file Image_5.jpg]

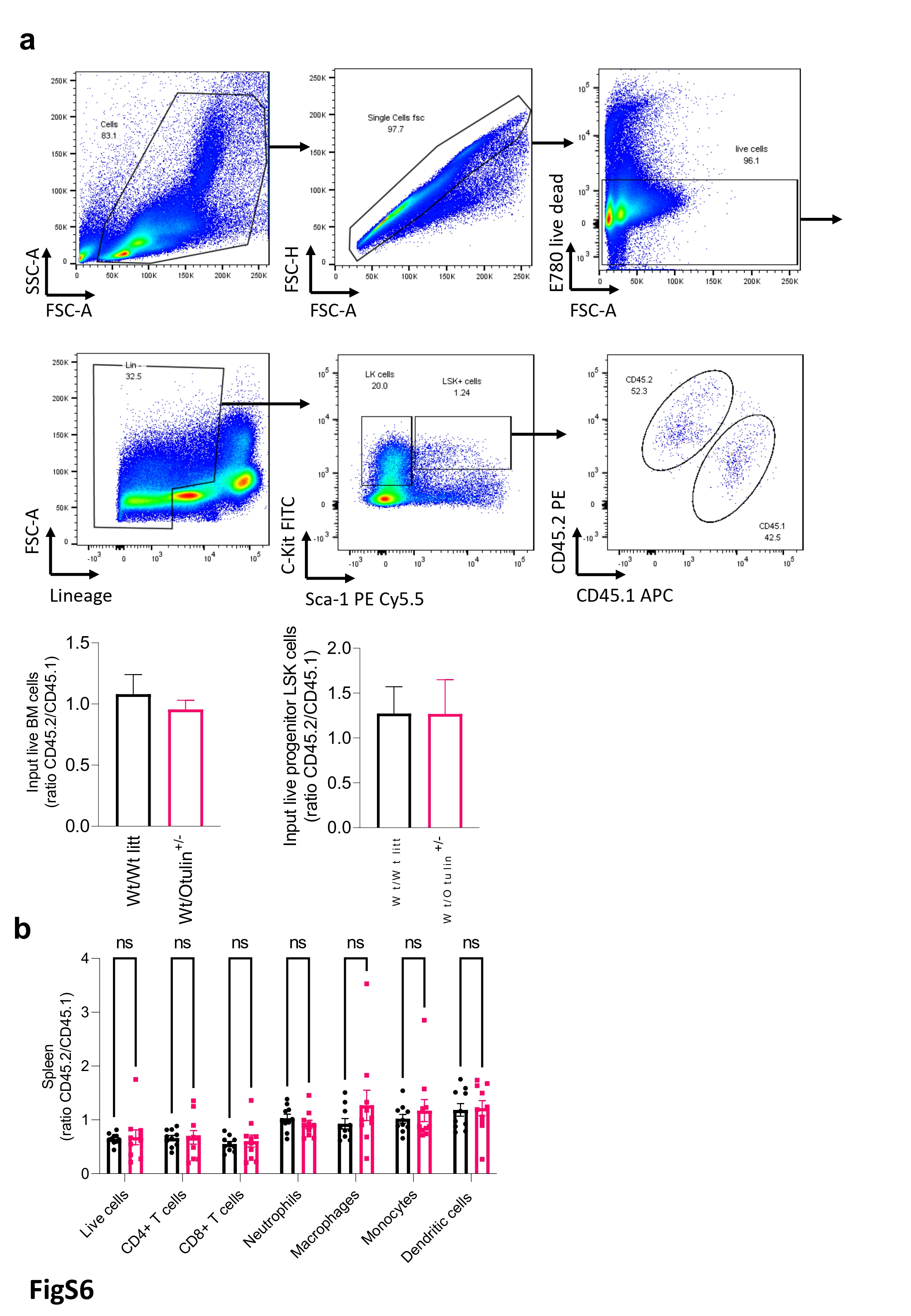

Supplement: Supplementary Figure 6 — Assessment of mouse chimerism after mixed bone marrow transplantation. (A). Gating strategy for bone marrow samples (A) and input ratio determination based on relative CD45.2 and CD45.1 counts from live CD45+ and progenitor cells (live CD45+ Lineage – cKit+ Sca-1+) cells. (B). Chimerism assessment 3 months after transplantation in lymphoid (T cells) and myeloid subsets (CD64+ monocytes and macrophages) in spleen, n=2 independent experiments, n=9-10 mice in each group. Bars represent mean ± SEM. [file Image_6.jpg]
